# The use of remote microphone systems in unilateral hearing loss: a preliminary study among Brazilian children and teenagers

**DOI:** 10.1590/1678-7757-2018-0744

**Published:** 2019-11-04

**Authors:** Maria Fernanda Capoani Garcia Mondelli, Regina Tangerino de Souza Jacob, Heitor Marques Honório

**Affiliations:** 1 Universidade de São Paulo, Faculdade de Odontologia de Bauru

**Keywords:** Unilateral hearing loss, Children, School, Remote microphone system

## Abstract

Due to the large number of individuals with Unilateral Hearing Loss (UHL) and the recommendation to use hearing assistive devices, studies are required to define possibilities of intervention for this population. Objective: To evaluate the performance of the Remote Microphone System (RMS) in children with UHL. Methodology: Prospective clinical study with a convenience sample. Eleven children (mean age of 9.2 years) with severe and profound sensorineural UHL, hearing aid users and enrolled in regular schools participated in the study. They were evaluated using the Hearing in Noise Test (HINT), the Classroom Participation Questionnaire (CPQ), and the Sustained Auditory Attention Ability Test (SAAAT) with RMS. Results: HINT results were analyzed using variance to three criteria of repeated measures, which revealed differences between intervention, position, and time factors and significant interaction between these three factors. The comparative analysis of the results from CPQ showed significant differences in the statistical t-test (p=<0.001) for all subscales. The analysis of variance at two repeated measures criteria used in the study of SAAAT revealed a difference between intervention and time, and both interacted significantly. Conclusion: The RMS associated with a hearing aid was effective for individuals with UHL.

## Introduction

The difficulties faced by children with unilateral hearing loss (UHL) are an essential demand in hearing health services. No consensus is reached on the treatment to this problem.

Nontreatment of UHL is considered a standard of care assistance in many parts of the world. Historically, doctors and researchers believed children with this type of hearing loss could have normal development since they had only one compromised ear. However, in the late 1980s, researchers began to detect significant academic problems in this population. Since then, studies have suggested children with UHL have a significant risk of speech, language, academic, and behavioral delays.^1^^,^^2^^,^^3^

With identification and early intervention, parents learn about the impact of hearing loss on child development. In Brazil, this became possible after the approval of Law 12.303 of August 2, 2010, which makes the Universal Newborn Hearing Screening Program mandatory.^4^ As a consequence, the age of detection of UHL in Brazil fell dramatically from school age to early childhood.^5^

In a recent study conducted in Ontario, Canada, with 20 children diagnosed with UHL, the mean age of identification was 4.6 months. Parents experienced significant uncertainty about the need to use amplification devices because the hearing loss and its potential impact on the child's development were considered minimal.^6^

In fact, among the communication difficulties caused by hearing loss, the ability to understand speech in noisy environments is a daily challenge,^2^ considering that most communicative situations occur in places where listening is hampered by the presence of competitive noise.

In classroom, understanding is critical for the child to master academic skills, such as determining the main idea, following directions, answering questions, and participating in class discussions. A sensory impairment can affect the concentration, grammatical and lexical knowledge, working memory, cognition, past experiences, and mental and physical state.^7^

Kuppler, et al.^8^ (2013) carried out a systematic review with articles from 1986 to 2012 regarding the academic performance of children with UHL. They observed hearing deprivation encompasses not only difficulty understanding speech in noisy places, but also exhaustion, self-esteem problems and, consequently, the need for greater effort to sustain auditory attention.

In another study, sustained auditory attention was evaluated in 90 children aged from 7 to 11 years. The children were separated in three groups, with 30 children each: normal hearing; diagnosed with mild bilateral conductive hearing loss; and diagnosed with mild bilateral sensorineural hearing loss. The authors concluded that sensorineural hearing loss causes greater impairment of sustained auditory attention when compared with conductive hearing loss.^9^

This ability can also be evaluated according to the period of the day and the type of school attended by the child. Picolini, et al.^10^ (2010) carried out a prospective study with 50 children of both sexes aged 7 years with normal hearing, without learning or behavioral complaints, and without complaints of attention difficulties. Participants underwent SAAAT (Sustained Auditory Attention Ability Test). The study concluded that children assessed in the afternoon and children that studied in public schools had a poorer performance in sustained auditory attention ability.

To initiate the rehabilitation process, hearing aids (HAs) are usually the first choice between electronic devices, minimizing the negative effects of hearing impairment on communication. However, HAs have one or more microphones that receive all sound signals, not just speech signals. For this reason, using this device may not guarantee a good signal-to-noise ratio, especially when, in addition to the background noise, other factors interfere with this relation, such as the distance of the sound source and/or reverberation.^2^^,^^6^^,^^8^

The development of HAs is based on studies on the spectral characteristics of speech and the results of perception tests with real or synthesized speech signals to provide acoustic information about speech signals that are more complete and closer to reality.^11^ For decades, manufacturers of sound amplification devices have been studying and developing technologies that can improve signal-to-noise ratio. The remote microphone system (RMS), which acts as a wireless microphone for the HA, is among the main features.

The recommendations of the American Academy of Audiology (AAA)^12^ state that the decision to recommend and fit a sound amplification device for this population should be made individually, considering several factors, such as the child's type of hearing loss, development, communication, education, and family preference.

Since the 1980s, scientific studies on the consequences of UHL have increased. However, studies about the intervention process in this population are scarce. Thus, one suggested the use of bilaterally adapted RMS could favor speech perception, classroom behavior, and sustained auditory attention.

## Objective

To evaluate the performance of the RMS in children with UHL and users of HAs, concerning speech perception, sustained auditory attention, and classroom participation.

## Methodology

A prospective clinical study, using a convenience sampling method, was conducted after the approval of the Research Ethics Committee (44955015.9.0000.5417) and TRIAL: RBR-5PGXYS, and after the agreement of parents or guardians, who signed the Informed Consent Form, and the consent of the individuals who participated in the investigation.

To participate in the study, the children should meet the following inclusion criteria:

– Diagnosed with severe or profound unilateral sensorineural hearing loss - UHL of 45 dB or higher (.5, 1.0, 2.0 and 4.0 kHz) in the affected ear and limits no worse than 15 dB (.5, 1.0, 2.0 and 4.0 kHz) in the normal hearing ear (World Health Organization).
– Effective users of HAs for at least one year, with daily use of 8 h confirmed by data logging and monitored periodically by professionals of the clinic.
– No previous experience with RMS.
– Enrolled in regular school.
– Aged between 5 and 17 years - according to the national criteria for free acquisition of the RMS.^13^
– After adapting to regular use of the RMS (school period) for three months for the second application of the procedures.^14^

The study was carried out with 11 children (7 female and 4 male), aged between 7 and 11 years, with severe unilateral sensorineural hearing loss (2 children) and profound congenital hearing loss (9 children). Six children had hearing loss in the left ear and five in the right ear. The mean age of detection of UHL was 4.4 years.

The children used the Naida III UP Phonak HA and were fitted with a remote microphone system from the same company connected by a wireless coupler. To verify the electroacoustic characteristics of the device, the Audioscan Verifit® equipment (Etymonic Design, Dorchester, Ontario, Canada) was used to verify transparency. As defined by the AAA,^15^ transparency is the condition in which equal inputs to the wireless and local microphones generate equal outputs from the hearing device.^15^

After the RMS fitting and verification, participants underwent the procedures in the following order:

### Hearing in Noise Test (HINT)

**Figure 1:**
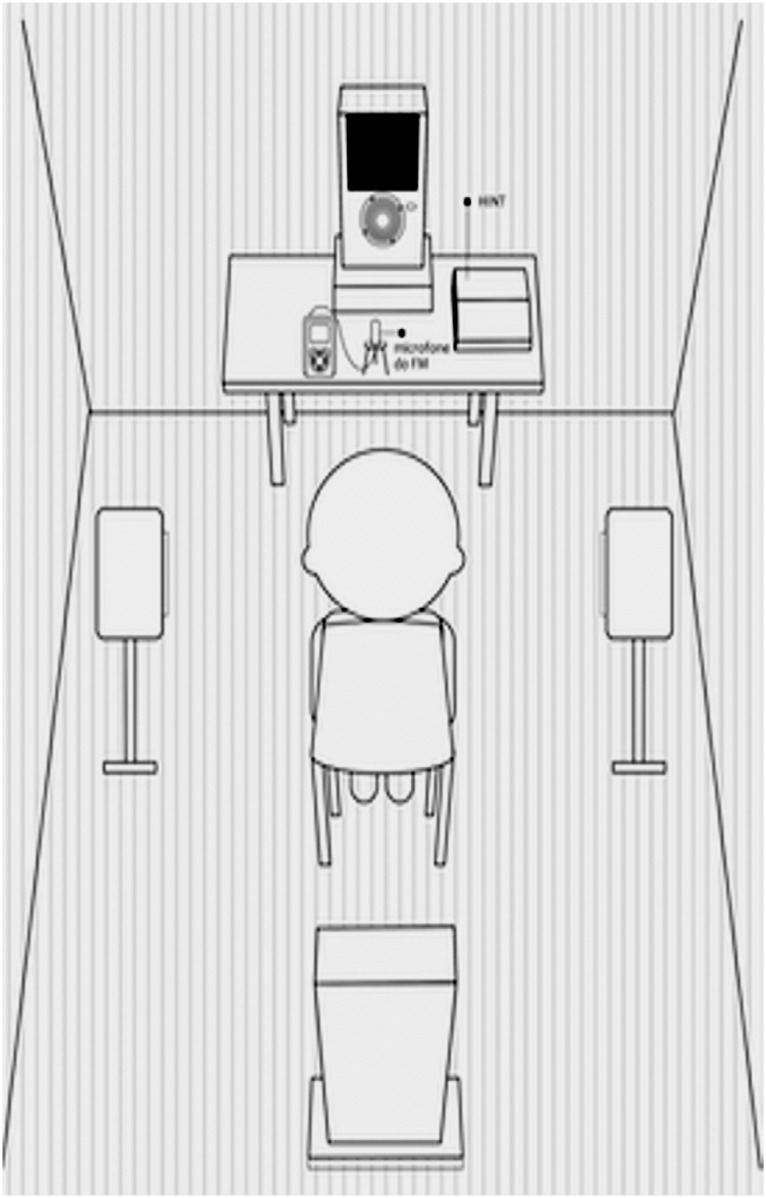
Positioning for HINT

The test was conducted in free field conditions in a sound-treated room, according to the HINTPro 7.2 Audiometric System operating instruction manual. HINTPro is an equipment with an interface connected to a computer. The specific software for the test must be installed on the computer, and the free field stereo loudspeakers must be coupled to the HINTPro 7.2. Each list of 20 sentences was applied in the situations: quiet (Q), noise front (NF), noise right (NR), noise left (NL), and compound noise (CN). The sentences were presented at 0---0° azimuth. The presentation level was initially set at 45 dBA and varied in steps of 4 dB and 2 dB according to the correct repetition of the level. The competitive noise was introduced at 0-0°, 0-90°, 0-180°, and 0-270° azimuths at a fixed intensity of 65 dBA.^16^ The lists of sentences and the order of noise were selected and randomly presented. The score was expressed in dB, representing the S/N ratio limit. According to the AAA^15^ recommendation for the use of the RMS, the Noise Back (NB) was positioned at 180° (Figure 1).

A negative S/N ratio indicates greater difficulty in the test and better performance of the individual. The more negative the ratio, the greater the difficulty, because the signal is emitted below the noise intensity.

### Classroom Participation Questionnaire (CPQ)

The CPQ is a subjective assessment tool that provides a situational analysis of student participation in classroom. It is a student-rated measure that contains 28 auditory situations divided into four subscales: (1) Understanding Teachers; (2) Understanding Students; (3) Positive Affect; (4). Negative Affect.

In each scale, the student scores choosing between 1 (almost never); 2 (sometimes); 3 (usually), and 4 (almost always).^17^ Higher scores are desirable except for the “Negative Affect” scale, in which the inverted score is the expected score.

Students took the test individually. They could read the items independently or have the items read or signed for them by the researcher, but all participants completed the questionnaire alone. Participants completed this questionnaire on the day of the RMS fitting and three months after. The RMS was used daily in classroom.

### Sustained Auditory Attention Ability Test (SAAAT)

The SAAAT is based on the Auditory Continuous Performance Test (ACPT), which is clinically used to measure auditory attention, and was performed in the free field.^18^ It is a method of evaluating the child's ability to listen to auditory stimuli over an extended period and to respond only to a specific stimulus. It consists of binaural and dichotic presentation of 21 monosyllabic words randomly rearranged and repeated to form a list of 100 words, with 20 occurrences of the target word “no”. This list, recorded on CD, was presented six times uninterruptedly, thus totaling 600 words, lasting approximately 10 min.

To determine the performance in this test, the children were instructed to raise their hand upon hearing the target word “no”. Two types of errors were considered: inattention error, when the child did not raise the hand in response to the target word, and impulsiveness error, when the child raised the hand in response to another word other than “no”. The score was calculated adding the number of inattention errors to the number of impulsiveness errors.

Vigilance was estimated by adding the number of correct answers for the word “no” during the sequence of the six presentations. The estimate was necessary to verify the decline in vigilance, i.e., the decline in the attention that occurred during the vigilance task, obtained by estimating the number of correct answers for the word “no” in the 1^st^ presentation and the number of correct answers for the 6^th^ presentation. The difference between these two numbers is called “decreased vigilance.”

To verify the better form of fitting of the RMS, the evaluations were performed under the following conditions: (A) only with HA (hearing aid); (B) HA in the ear with hearing loss and RMS in both ears; (C) HA in the ear with hearing loss and RMS in the ear with normal hearing; (D) HA and RMS only in the ear with hearing loss. The children used the devices in the different conditions in classroom for 15 days each.

To avoid learning effect on the evaluations, the different conditions were applied with a 15-day interval, according to Keith^19^ (1994), and the Latin Square Design was used to present the children's evaluation conditions.

### Statistical analysis

Statistical analysis was initiated by examining the data using the normality test (Shapiro-Wilk) and the variance homogeneity test (Levene). If normal distribution and homogeneity of variance are not considered significant, parametric tests would be used; otherwise, nonparametric equivalent statistical tests would be used.

For the HINT data, there was a normal distribution of the data and homogeneity of the variances; therefore, the two-way repeated measures ANOVA (factors: Intervention and Time) and the Tukey's test were performed for multiple comparisons.

For the results from the CPQ, the comparative analysis of the “teacher comprehension” and “peer comprehension” showed normal distribution of the data and homogeneity of the variances, so the Student's t-test was used. For the “positive and negative aspects,” data were not normally distributed, so the nonparametric Mann-Whitney test was used.

For the SAAAT data, parameters of normality and homogeneity were verified; therefore, they were submitted to the test of analysis of variance for two criteria of repeated measurements.

For all cases, a significance level of 5% was adopted.

## Results

Since HINT is a sentence repetition test, age is not expected to influence the results. The results were studied through analysis of variance for two criteria of repeated measures, in which differences between intervention and time factors were observed. However, the same test revealed a significant interaction between these two factors, and the Tukey's *post hoc* analysis allowed the verification of these differences, shown in Table 1.

**Table 1:** Results obtained in the HINT in the different positions and with different configurations of the HA and the RMS

Intervention	Time	Mean ± SD
Quiet		
A	1^st^ evaluation	40.33 ± 2.17^cd^
	3 months	40.51 ± 1.35^d^
B	1^st^ evaluation	35.97 ± 1.75^ab^
	3 months	34.18 ± 2.33^e^
C	1^st^ evaluation	38.24 ± 1.78^bcd^
	3 months	36.18 ± 2.75^ae^
D	1^st^ evaluation	37.78 ± 1.57^abc^
	3 months	37.20 ± 1.14^ab^
	Noise Front	
A	1^st^ evaluation	1.15 ±0.73^c^
	3 months	1.15 ±0.73^c^
B	1^st^ evaluation	-2.05 ±0.89^a^
	3 months	-1.52 ±1.23^a^
C	1^st^ evaluation	0.02 ±1.33^bc^
	3 months	-2.11±0.84^a^
D	1^st^ evaluation	-1.39 ±1.30^ab^
	3 months	-1.23 ±1.74^ab^
	Noise Right	
A	1^st^ evaluation	0.18 ±1.38^b^
	3 months	0.18 ±1.38^b^
B	1^st^ evaluation	-2.95 ±1.07^a^
	3 months	-3.85 ±1.88^a^
C	1^st^ evaluation	0.65 ±1.18^b^
	3 months	-2.00±1.38^a^
D	1^st^ evaluation	-3.81 ±2.25^a^
	3 months	-5.65 ±1.17^c^
	Noise Left	
A	1^st^ evaluation	-0.13 ±2.4^c^
	3 months	-0.13±2.4^c^
B	1^st^ evaluation	-1.81 ±3.07^ab^
	3 months	-3.58 ±2.63^a^
C	1^st^ evaluation	-1.82 ±2.06^ab^
	3 months	-1.67±2.63^ab^
D	1^st^ evaluation	-2.34 ±2.8^ab^
	3 months	-4.59±1.5^a^
	Compound Noise	
A	1^st^ evaluation	-0.89 ±1.16^a^
	3 months	-0.89 ±1.16^a^
B	1^st^ evaluation	-1.80 ±2.4^ab^
	3 months	-3.11 ±0.82^b^
C	1^st^ evaluation	-0.83 ±1.44^a^
	3 months	-3.22±1.03^b^
D	1^st^ evaluation	-0.86 ±1.42^a^
	3 months	-3.28 ±1.10^b^
	Noise Back	
A	1^st^ evaluation	-1.28 ±2.08^ad^
	3 months	-1.28 ±2.08^ad^
B	1^st^ evaluation	-4.15 ±1.3^bc^
	3 months	0.26 ±2.1^d^
C	1^st^ evaluation	-5.63 ±1.11^b^
	3 months	-3.22±1.03^b^
D	1^st^ evaluation	-2.76 ±1.8^ac^
	3 months	-3.23 ±1.03^ac^

* Different letters indicate statistically significant differences in the comparison between the groups (Two Way Repeated measures ANOVA and Tukey's test) in each position and each configuration of the HA and the RMS. If at least one letter is coincident in the comparison between groups, then there will be no statistically significant differences between these groups.

A= Hearing Aid; B= Hearing Aid and Remote Microphone System bilaterally adapted; C= Hearing Aid and Remote Microphone System in affected ear; D= Hearing Aid adapted in the affected ear and Remote Microphone System in the normal ear

Improvement in the responses in all HINT situations using the RMS was observed.

**Figure 2:**
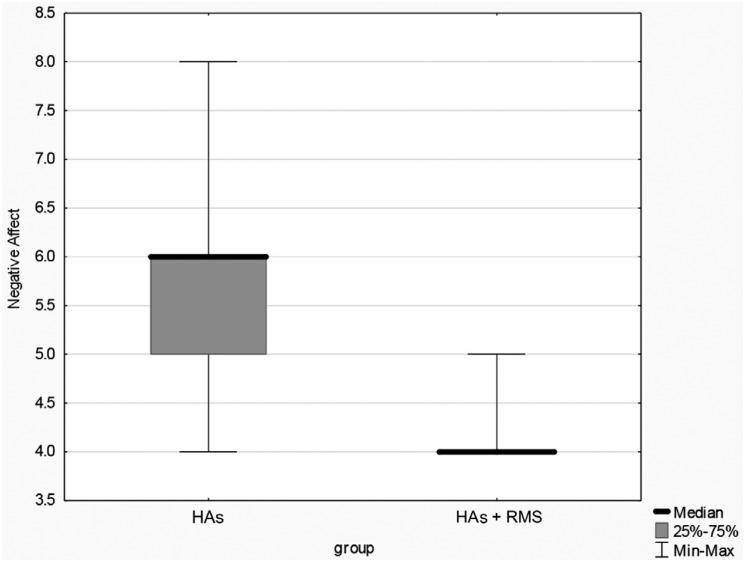
Positive aspects

**Figure 3:**
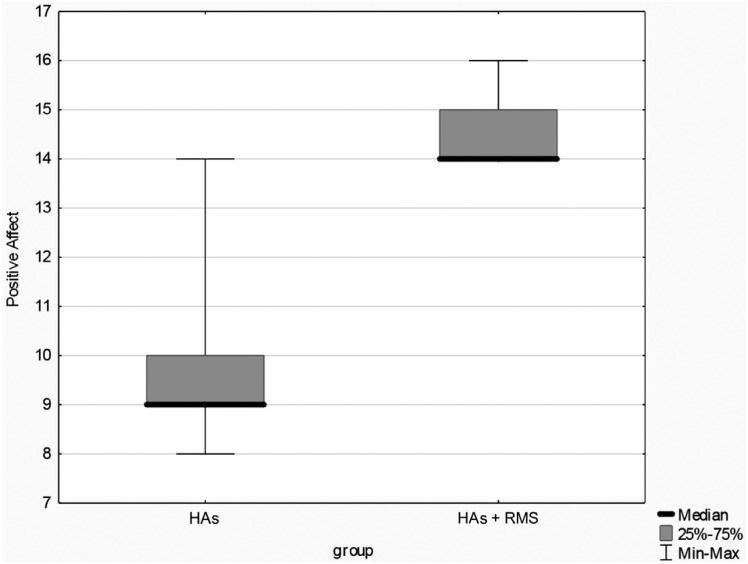
Negative aspects

The comparative analysis of the results from the Classroom Participation Questionnaire, with and without the use of the RMS, showed significant differences in the statistical t-test (p=<0.001) for the subscales “teacher comprehension” and “peer comprehension”, since (Shapiro-Wilk test) homogeneity of variances (Levene's test) were observed in these cases. In the “positive aspects” and “negative aspects,” data were not normally distributed (Shapiro-Wilk test). The use of the nonparametric Mann-Whitney test indicated a statistically significant difference between groups (Tables 2, and Figures 2 and 3).

**Table 2:** Results of the "Understanding Teacher" and "Understanding Students" subscales of the CPQ

	Subescales	Average± dP	p
HA	Understanding	10.7±1.2^a^	p <0.001
HA and RMS	Teacher	13.9±1.4^b^	
HA	Understanding	8.8±1.8^a^	p <0.001
HA and RMS	Students	14.9±0.9^b^	

* Different overlapping letters indicate statistically significant differences in the comparison between the groups (Student's t-test).

HA = Hearing Aid; RMS = Remote Microphone System

The classroom participation of the children evaluated presented significant results using the RMS in all categories.

The analysis of variance at two criteria of repeated measures used in the study of the results of the SAAAT showed a difference between intervention and time, and both interacted significantly. Table 3 indicates the results for the total score.

**Table 3:** Results of the total scores obtained in the SAAAT

Intervention	Time	Average± dP
A	1^st^ evaluation	17.09±6.39^e^
	3 months	17.82±5.76^e^
B	1^st^ evaluation	6.27±4.20^abcd^
	3 months	4.27±3.47^abcd^
C	1^st^ evaluation	8.27±5.02^bd^
	3 months	5.73±4.43^ac^
D	1^st^ evaluation	8.09±4.13^cd^
	3 months	4.00±3.35^ab^

* Different overlapping letters indicate statistically significant differences in the comparison between the groups

A = Hearing Aid; B = Hearing Aid and Remote Microphone System fitted bilaterally; C = Hearing Aid and Remote Microphone System in affected ear; D = Hearing Aid fitted in the affected ear and Remote Microphone System in the normal ear

The SAAAT presented statistically significant results in interventions C and D.

## Discussion

The literature determines the use of the RMS as assistive technology for children with hearing loss, especially in classroom.^15^ Patient-related behaviors with UHL are still inadequately established, hindering professionals in the area from making decisions.

The group studied presented a diagnosis of sensorineural hearing loss with severe (two children) and profound (nine children) degrees, without the prevalence of sex or ear. The mean age of detection of UHL was 4.4 years, as in the study in Ontario (Canada),^6^ in which the mean age of hearing loss was 4.6 years. The Universal Newborn Hearing Screening Program is resulting in the interest and the search of the families for early intervention, justifying the significant demand of the hearing health services.

The AAA protocol^12^ indicates that the fitting of sound amplification devices in children with UHL should be considered based on evidence of language development delays and academic difficulty. The recommendation depends on the child's needs, the family's motivation, and the clinical professional's judgment, and should be made individually.

The participants were fitted with HAs and used them for at least 8 h, suggesting that hearing loss affects the children's development in some aspects, such as school, attention, sound localization, and speech comprehension. For inclusion in the project, the children used the RMS regularly in classroom. The effectiveness of the RMS depends on its periodic verification.^20^

This study verified the effectiveness of the RMS through the performance of children with UHL in tasks of speech perception, classroom participation, and sustained auditory attention.

### Speech perception

Speech perception was evaluated through the HINT Brazil^16^. The responses were obtained in quiet (Q) and in noise generated in different positions: noise front (NF), noise right (NR), noise left (NL), noise back (NB), and composite noise (CN). The test was also conducted with HA alone and associated with the RMS in three different conditions: bilaterally, in the ear with hearing loss and in the ear with normal hearing (Table 1). After statistical analysis, no significant difference between the RMS positioning conditions was observed. These results are similar to those found by Updike^21^ (1994) and Tharpe, et al.^22^ (2004), which report improvement in speech recognition in children with UHL in a situation of noise and silence using the RMS.

Regardless of the HINT application, the software itself uses the composite noise (CN), which is a weighted average of the NF/NR/NL conditions for the free field application. The results from the CN (Table 1) after three months using the devices showed values between −3.1 and −3.3. The values were lower than −5.9, found in the group of 21 normal-hearing children and teenagers, aged between 7 and 14 years, evaluated by Jacob, et al.^23^ (2011). However, these values were closer to 4.83 from the evaluation of adults in the same test.^24^

The study^23^^–^^25^ reported that the best expected responses are in the noise and speech condition separated by 90°, with speech at 0° in front of the individual and the noise at 90° to the right or left. In this study, the best values were obtained with the RMS only in the ear without hearing loss, with a mean of −5.6 for NR and −4.5 for NL.

The NR and NL results (Table 1) were better with the system attached to the ear without involvement, with speech at the front and noise at the side of the ear with hearing loss, justifying the data similar to those surveyed in subjects with normal hearing.^23^^,^^24^

Regarding NF, the children studied presented lower rate of improvement by using the RMS, presenting a response value of −2.6 with the use of HA and the RMS positioned in the affected ear. This is the best result concerning the speech perception in noise front (Table 1). These results agree with those of previous studies^23^^,^^24^^,^^26^ that evaluated children and adults.

The value of −6.04 from the NB with HA and RMS in the affected ear (Table 1) was similar to the mean of −6.2 obtained by Sbompato, et al.^24^ (2015) with normal-hearing children. This similarity suggests using the devices allows the child to present speech perception in a situation of noise from behind similar to children without hearing loss. The AAA^15^ recommends that the RMS benefit checks be performed with NB, similar to the noises generated by classmates in a classroom situation.

In a quietness situation, the expectation is that the RMS increases the level of speech perception by at least 10 dB concerning only the speech perception with a HA.^15^^,^^27^ Previous studies with HA and RMS were performed in individuals with bilateral hearing loss, justifying the difference in perception in the group studied, with responses in 40 dBNA using HA and in 34 dBNA with the RMS coupled in both ears. In a study with 30 adults with UHL, the results of the HINT in quiet were 40 and 39 dBNA, with and without a HA^26^^,^, respectively. The results of this study were similar to other results found in the literature, such as the Hearing in Noise Test-Children and Nonsense Syllable Test.^28^

The HINT was used to evaluate children and teenagers with cochlear implants, with different box placements and stimuli. The results were significant when comparing the responses with and without the RMS.^29^ The benefit of the RMS is devoted to bilateral hearing loss and has also proved to be effective for UHL.

The awareness of the teacher of children with UHL in classroom is necessary. Dancer, et al.^30^ (1995) observed teachers gave lower grades to children with UHL - unaware of the hearing impairment - in all five SIFTERs (Screening Instrument for Targeting Educational Risk). In this study, children were effective users of HAs and, after the RMS were fitted, their teachers were instructed to use and take care of the device, recommending speech therapy support for the effective use of this technology.^31^

### Classroom participation

Children spend at least 4 h a day in school, totaling 20 h a week. Antia, et al.^32^ (2011) report that the student's ability to communicate and participate effectively in classroom with teachers and other students is a predictor of academic success for students with hearing loss. Children with hearing loss are increasingly likely to attend regular classrooms due to early identification/intervention and better access to auditory information. Griffin^33^ (2015) points out that academic difficulties and failures in the UHL population are more generalized than initially thought; thus, intervention may provide more favorable results for school performance.^8^

Questionnaires are considered valuable instruments to verify the classroom performance of these children. The CPQ was used in this investigation.^17^ Children's classroom participation presented significant results with the use of the RMS in all categories (Tables 2, and Figures 2 and 3), also indicating an improvement in the subscales of the questionnaire. Children found it easier to understand the teacher and classmates and felt more confident. A similar result was obtained in a study^17^ that applied the questionnaire in a group of 15 children and teenagers using a cochlear implant or HA, before and after the RMS fitting.

According to researchers,^34^ early childhood is the most important listening period. However, children often face more difficulty in noise-challenging acoustic environments. The group studied here used the HA associated with the RMS in classroom. The analysis of the results suggests the noise existing in school, which interferes with speech comprehension and student participation within the group, was minimized by the devices. Table 2 shows the mean values of the student's comprehension of the teacher's speech (13.9) and their classmates’ speech (14.9), which are very close to the maximum possible value (16).

“Positive Affect” in the CPQ refers to the student speaking in classroom, speaking with the teacher and participating in group discussions. It showed significant improvement in the results (Figure 2), suggesting that group participation may contribute to children's social and emotional well-being and to a better academic performance, according to the conclusion of studies carried out with the CPQ.^32^^,^^35^ The “negative affect” scale refers to frustration with group communication and with the teacher. It is the only scale in the CPQ in which lower scores are desirable. In this study, the participants obtained minimum score (4) (Figure 3), indicating absence of difficulties in these situations.

### Sustained auditory attention

The auditory attention of schoolchildren can be influenced by auditory alterations, causing impairment in attention and comprehension skills, and, consequently, compromising the performance and learning of these children.^10^ For health and education professionals, understanding the effects of these variables on attention skills is essential, since sustained attention and vigilance, when altered, cause difficulty in concentrating on tasks, impairing the child's development and learning.^36^

In this study, the SAAAT evaluated the change in the responses of sustained attention after using sound amplification devices, assuming the importance of attention to the learning process, especially in classroom with presence of noise. Considering that the total score in the SAAAT is quantified according to the number of impulsiveness and inattention errors, the participants presented a mean value of 4.0 after three months using the RMS (Table 3), with better results than found (7.7)^9^ in children with normal hearing. This result confirms that the RMS influenced positively the children's sustained attention.

This result can be justified since a favorable signal-to-noise ratio facilitates attention to tasks and improves response time. It happens because the children have a longer time to focus and concentrate on the stimulus when the teacher's speech becomes clearer, while ignoring the competitive stimulus.

According to the literature, younger children have a limited attention span and, as they develop it, the internal processing mechanism changes, increasing this capacity. Therefore, older children, aged 11 years, perform better than younger children in this ability.^37^^,^^38^ Although the mean age of the children evaluated in this study was 9.2 years, the results after three months of use of the RMS were compatible with the responses of children with normal hearing in the 11-year age group.^39^

The participants used the HA/RMS in classroom according to the proposal of the study, with results with poor statistical significance. In the HINT and SAAAT evaluations, the children reported that classroom situations became easier to understand and, at the end of the period, they were less tired (less auditory effort). Informally, the children reported higher concentration to receive the teachers’ explanation and to better understand the subjects. The children also reported that asking the teacher to repeat what was just said during dictation activity was no longer necessary.

In a systematic review of sound amplification devices for unilateral sensorineural hearing loss of severe and profound degrees in adults, the authors suggest researchers conduct further studies on effectiveness of sound amplification devices for this type of hearing loss, thus supporting efforts for new evidences.^40^ Finally, it should be recognized that UHL is a crucial problem for school-aged children and that increasing studies for interventions that benefit this population is necessary.

The results of this investigation evidence that the RMS should be indicated for children with UHL, mainly for use in classroom, regardless of the fitting condition.

This investigation was carried out with 11 children, because it is a study that requires periodic returns to the clinic, control of the use of devices and availability of equipment. Thus, the sample number is small, similar to international surveys conducted with children with UHL.^3^^,^^6^^,^^30^^,^^33^ We highlight that no previous studies on UHL have similar methodology and literature on this subject is limited.^8^

## Conclusion

The research participants presented superior results using the RMS in evaluations of speech perception, sustained auditory attention, and classroom participation, regardless of the fitting condition, suggesting the effectiveness of this device associated with a hearing aid for children with unilateral hearing loss.
